# Anxiety levels during COVID 19 pandemic in primary and secondary doctors in UK

**DOI:** 10.1192/bjo.2021.186

**Published:** 2021-06-18

**Authors:** Dr Shweta Mittal, Abdalla Abouebeid

**Affiliations:** 1 Nottinghamshire Healthcare NHS Foundation Trust, Bassetlaw Mental Health Services; 2 The St.Vincent Practice

## Abstract

**Aims:**

The study aims to examine the severity of anxiety in primary and secondary doctors in the UK during first wave of COVID-19 pandemic.

**Method:**

An online General Anxiety Disorder-7 (GAD7) survey was distributed during the first wave of COVID-19 pandemic (April-May 2020) to doctors in primary and secondary care in the UK. Seven closed-ended questions were included in the questionnaire. Respondents were to indicate how frequently they experienced specific issues in the previous fortnight: Feeling nervous, anxious, or on edge; being unable to stop or control worrying; worrying too much generally; trouble relaxing; being so restless that it's hard to sit still; becoming easily annoyed or irritable, feeling afraid of something awful happening. Participants were required to tick one of four choices for each of the seven parameters - not at all (0), several days (1), more than half the days (2) and nearly every day (3). A person with minimal or no anxiety will score less than 5. The survey was anonymous and circulated in professional online doctors' forums. Participation was voluntary and no incentives were given.

**Result:**

273 completed surveys were received; 120 doctors were in primary care and 153 were in secondary care. Average GAD7 score was 6.4 in primary care and 7.9 in secondary care. 57% of primary care doctors and 66% of secondary care doctors reported score of 5 or more, representing at least mild anxiety symptoms. 22% doctors in primary care and 31% doctors in secondary care reported GAD7 score of 10 or more, indicating moderate to severe anxiety. One in ten doctors in both primary and secondary care reported severe anxiety due to the ongoing COVID-19 pandemic.

**Conclusion:**

The finding of more anxiety in secondary care doctors might be because general practitioners could resort early in the pandemic to remote consultations along with inadequacy of resources, greater exposure to suffering/deaths of patients and colleagues in hospital and perceived risk of catching COVID-19 infection.

Results are limited due to relatively low numbers and it would be useful to replicate this study on a larger scale. Doctors are less likely to acknowledge their mental health difficulties due to stigma associated with mental health.

Many employers have psychological support systems in place for their staff, but it is questionable if affected individuals are willing to receive this support. This paper; therefore, calls for creating open anonymous platforms for professionals to get access to appropriate support to address their anxiety.

